# Junctional Epidermolysis Bullosa: Allelic Heterogeneity and Mutation Stratification for Precision Medicine

**DOI:** 10.3389/fmed.2018.00363

**Published:** 2019-01-29

**Authors:** Irina Condrat, Yinghong He, Rodica Cosgarea, Cristina Has

**Affiliations:** ^1^Department of Dermatology and Venerology, Medical Center - University of Freiburg, Faculty of Medicine, University of Freiburg, Freiburg, Germany; ^2^Department of Dermatology, Iuliu Hatieganu University of Medicine and Pharmacy, Cluj Napoca, Romania

**Keywords:** junctional epidermolysis bullosa, collagen XVII, *COL17A1*, mutation, premature termination codon, therapy

## Abstract

Junctional epidermolysis bullosa (JEB) is a hereditary blistering disease caused by reduced dermal-epidermal adhesion due to deficiencies of one of the proteins, laminin-332, type XVII collagen, integrin α6β4 or integrin α3. Significant progress has been achieved in the development of therapies for EB, such as bone-marrow transplantation, local or systemic injections with fibroblasts or mesenchymal stromal cells, readthrough of premature termination codons, or exon skipping. These were tailored in particular for dystrophic EB, which is caused by type VII collagen deficiency and have not yet reached broad clinical practice. Recently, pioneering combined gene and stem cell therapy was successful in treating one boy with junctional EB. Beside these exclusive approaches, no specific therapy to amend the major clinical features, skin and mucosal blistering and non-healing wounds is available to date. Here we extend the mutational spectrum of junctional EB, provide a stratification of *COL17A1* mutations and discuss potential molecular therapeutic approaches.

## Introduction

Inherited epidermolysis bullosa (EB) is a group of genetic diseases characterized by skin fragility and is caused by mutations in the genes encoding different proteins with roles in cell adhesion. Junctional EB (JEB) is a subtype of EB in which dermal-epidermal adhesion is reduced, due to deficiencies in one of the proteins laminin-332, type XVII collagen, integrin α6β4 or integrin α3 (1).

Type XVII collagen (also known as ERED, BP180, BPA-2, BPAG2, LAD-1, BA16H23.2) is expressed by basal epidermal keratinocytes and plays an important role in cell-matrix interactions as a transmembrane component of the hemidesmosomes. Structurally, type XVII collagen is a homotrimer, consisting of three collagen alpha-1(XVII) chains, each with a molecular weight of 180-kDa. It is a type II protein, which spans the cell membrane with two domains, an endodomain toward the cytosol and an ectodomain toward the lamina densa of the basement membrane. It links, together with integrin α6β4 and CD151, the inner hemidesmosomal plaque consisting of plectin and BPAG1 to the anchoring filaments buildup of laminin-332 (2). The primary, secondary and tertiary structures, post-translational modifications, as well as interactions and functions of type XVII collagen have been recently extensively reviewed (3, 4).

The main function of type XVII collagen as an adhesion molecule is assured by tight, but dynamic incorporation into hemidesmosomal multiprotein complexes in stratified, pseudostratified and transitional epithelia (e.g., skin, oral mucosa, ocular conjunctiva, epithelial basement membrane of the cornea, upper esophagus, transitional epithelium of the bladder). Besides this role in cell-matrix adhesion, type XVII collagen is responsible for maintenance of follicular stem cells, cell polarity, and migration.

Against this dogma, recent studies provide evidence for the presence of type XVII collagen between the basal keratinocytes, this type being known as the non-hemidesmosomal collagen XVII (4). Non-hemidesmosomal collagen XVII is not a part of the dermal-epidermal adhesion and after its discovery, questions emerged regarding its role. Molecular interactions and cross-talk with focal adhesions and the actin cytoskeleton may be implicated (5, 6). Watanabe and colleagues reported that reduction of non-hemidesmosomal collagen XVII in the interfollicular epidermis is involved in physiological aging, but further work is required in order to decode the exact mechanism (4, 5). Besides these findings, type XVII collagen is also found in the hair follicles stem cells and its proteolytic degradation grounds for age-related hair loss, making it a potential targeted candidate for age-related alopecia, explaining definitive hair loss in patients with JEB (7, 8).

**Figure 1:**
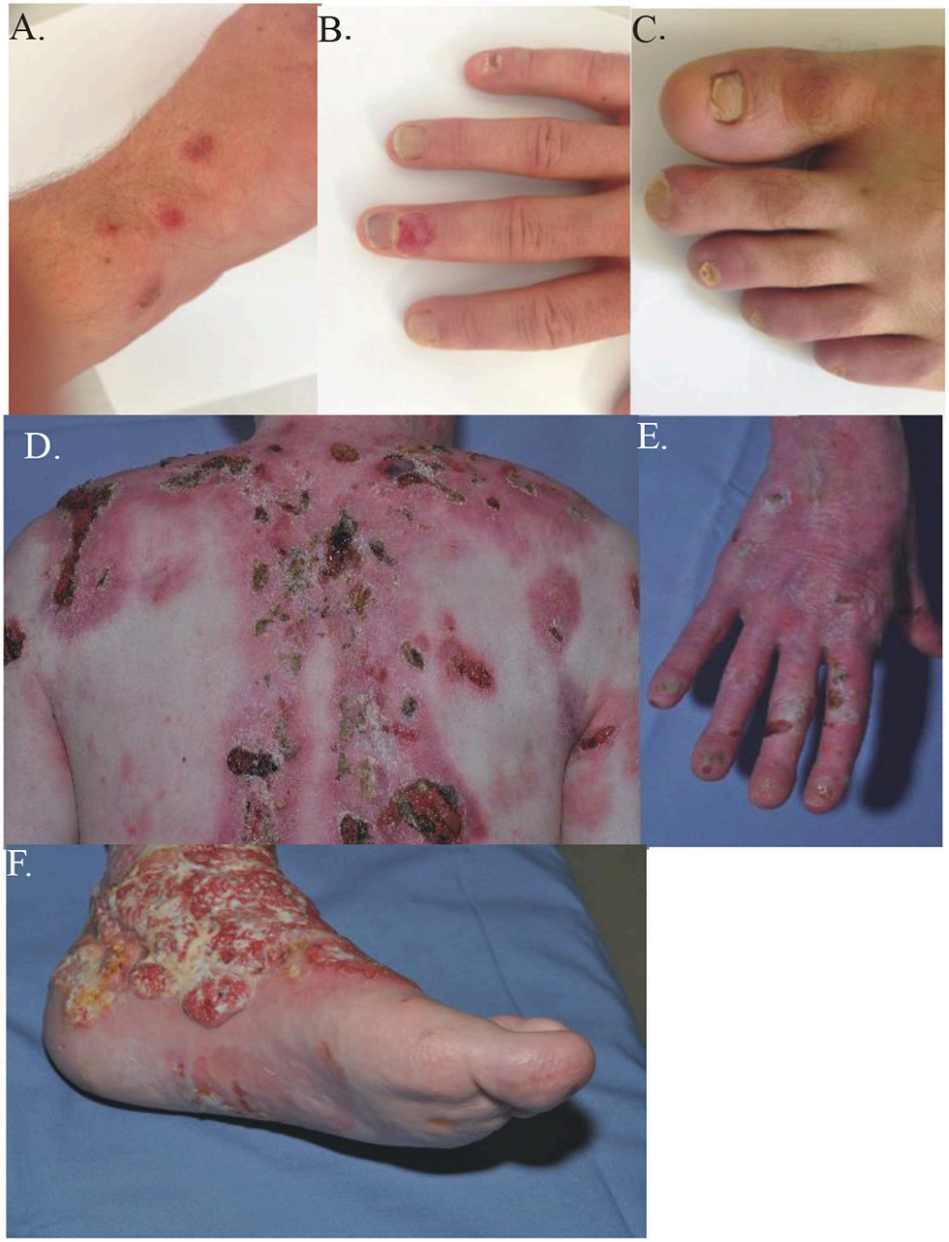
Clinical presentation and different phenotypes in JEB. The clinical manifestations of two JEB patients, both with *COL17A1* mutations. **(A–C)** Case 66, compound heterozygous with a missense mutation c.3908G > A and a deletion c.4100_4101delTT, mildly affected. **(A)** discrete erosions and blisters on upper hand; **(B)** nail dystrophy and crusts on the hand; **(C)** toe-nail dystrophy. **(D–F)** Case 68, a 45 y-old male compound heterozygous with a missense mutation c.2T > A and a deletion c.3164delT, severely affected. **(D)** Extensive crusts and scarring on the upper posterior thorax. **(E)** Nail dystrophy, erosions and crusts on the right hand. **(F)** Squamous cell carcinoma on the lower left leg.

Type XVII collagen is encoded by *COL17A1* which spans 52 kb of the genome and is located on the long arm of chromosome 10 (10q24.3). *COL17A1* consists of 56 exons and short introns. Mutations in this gene lead to a complete or partial loss-of-function of type XVII collagen in tissues and cause generalized or localized skin blistering, amelogenesis imperfecta, epithelial recurrent erosion dystrophy, alopecia and nail dystrophy (3). Although life expectancy is not reduced, patients with JEB due to *COL17A1* pathogenic variants experience extensive trauma-induced blistering resulting in multiple wounds that tend to heal slower with time, excessive caries, diffuse progressive irreversible alopecia, and have impaired quality of life (Figure 1).

Currently, EB research is focused on elucidating the disease mechanisms and development of therapies (9). Experimental therapeutic approaches include *ex vivo* gene therapy to correct *LAMB3* and *COL7A1* mutations in epidermal stem cells (10, 11), cell therapies for dystrophic EB, repurposed drugs with anti-inflammatory effects for dystrophic EB and EB simplex and topical agents aiming at improving wound healing. There is an urgent need for new treatments for JEB with *COL17A1* mutations for which no experimental therapies are presently available.

Extensive genetic testing and research has provided a comprehensive database (Human Gene Mutation Database^®^ Professional 2018.2) with over 100 distinct mutations encountered in *COL17A1*, but the database constant development is an ongoing task. The need for such mutational and patients databases is crucial for patients' counseling and prognosis. Development of therapies and selection of patients for clinical trials also depends on their individual mutations.

Here, we review our cohort comprising of 68 JEB patients with *COL17A1* mutations diagnosed over the past 15 years, report novel mutations, genotype-phenotype correlations and finally, discuss potential experimental therapies for these patients.

## Methods

The study was conducted according to the Principles of Helsinki and was approved by the Ethics Committee of the University of Freiburg. Written informed consent was obtained from the participants for the publication of the case reports and identifiable images included in this article.

### Mutation Detection

After informed consent genomic DNA was extracted from EDTA-blood using the Qiagen blood minikit (Qiagen, Hilden Germany). In most cases, mutation analysis was performed by bidirectional Sanger sequencing as reported before (12). Targeted next generation sequencing (NGS) of EB genes was performed since 2016 as described (13). In one case clinical exome analysis was performed (http://www.humangenetik-freiburg.de/).

### Cell Culture

Human primary keratinocytes were isolated from the skin of the patients and immortalized with the E6E7 genes as previously described (14). Keratinocyte lines were cultured at 37°C in 5% CO_2_ in serum-free medium containing epidermal growth factor and bovine pituitary extract (Invitrogen).

### RNA Isolation and RT-PCR

Isolation of total RNA from confluent cell monolayers was performed using the RNeasy Plus Kit (Qiagen, Hilden, Germany) according to the manufacturer's instructions. One Microgram of isolated RNA was used for cDNA synthesis with the First Strand cDNA Synthesis Kit (Thermo Fisher Scientific, Wilmington, USA) in a volume of 100 μl.

RT-PCR was then performed with 5 μl of the cDNA in a 50 μl mix containing 10x Puffer, nucleoside diphosphatase (dNTP), Taq DNA Polymerase (Sigma) and primers. Primers for *COL17A1* cDNA were F: TACCATGTACGTGTCAGGCC and R: TGATGCTGGACCACACATTG. The annealing temperature was calculated with UCSC *in-silico* PCR (https://genome.ucsc.edu/cgi-bin/hgPcr).

### Immunofluorescence Staining of Skin Sections

Biopsy technique, tissue processions, antigen-antibody interaction, and afterwards visualization were previously described (15).

### Protein Isolation and Immunoblotting

For protein extraction, cells were lysed on ice for 20 min in lysis buffer, proteinase inhibitors and phosphatase inhibitors and centrifuged for 20 min at 14,000 rpm. Extracts were subsequently heated to 95°C for 5 min. Bradford assay (BioRad) was used to determine the protein concentration. For collagen XVII immunodetection (case 61), 35 μg protein was run on a 8% SDS Page for 1.5 h and transferred to a nitrocellulose membrane at 300 mA for 1.5 h. For detection, collagen XVII-specific antibodies were used: NC16A, against epitope within the NC16A region, at a dilution of 1:1000 (16).

## Results

The results of the genetic and molecular analysis of the cohort are described below. Also, novel mutations, genotype-phenotype correlations, and immunofluorescence mapping are reported in Table 1.

**Table 1:** Mutational analysis, immunofluorescence mapping and genotype-phenotype correlations of the patients previously unpublished.

**Case**	**Mutations c.DNA level**	**Mutation Protein level**	**Immunofluorescence**	**Clinical phenotype (age)**
44	**c.4153C>T**	p.Q1385^*^	Not available	Not available
45	c.2861delG	p.G954Afs^*^112	Not available	Not available
49	c.2062C>T	p.R688^*^	Negative staining for collagen XVII	Severely affected with generalized blistering, nail dystrophy (age 1)
50	c.2407G>T	p.G803^*^	Reduced immunoreactivity for collagen XVII	Multiple blisters and erosions, nail loss, dental abnormalities (age 3)
51	c.[3487G>T;4319dup]	p.[E1163^*^;G1441Wfs^*^14]	Reduced immunoreactivity for collagen XVII	Mildly affected, nail dystrophy (age 1)
52	c.2237del	p.G746Afs^*^53	Reduced immunoreactivity for collagen XVII, almost negative	Severely affected, extensive blisters and erosion, nail loss (1 week old)
58	c.418_419delAG	p.S140^*^	Reduced immunoreactivity for collagen XVII, almost negative	Not available
59	c.[2237delG;3198C>T]	p.[G746Afs^*^53;S1066S]	Strongly reduced immunoreactivity for collagen XVII at the blister roof	Mildly affected
60	c.2062C>T	R688^*^	Negative staining for collagen XVII	Not available
61	**c.[1750C>T;3509-1G>C]**	p.[R584^*^;?]	Reduced immunoreactivity for collagen XVII at blister floor (Figure 3)	Mildly affected, nail dystrophy and loss (age 2) (Figure 3)
62	**c.2282_2283delGG**	p.G761Dfs^*^40	Reduced immunoreactivity for collagen XVII, almost negative	Not available
64	**c.[779delC;3569dupG]**	p.[P260Qfs^*^32;N1191Qfs^*^51]	Reduced immunoreactivity for collagen XVII at blister roof	Skin blisters, alopecia, nail loss
65	c.2062C>T	p.R688^*^	Negative staining for collagen XVII	Flaccid serous and hemorrhagic blisters, alopecia, poikiloderma, multiple hypopigmented scarred areas, mucosal involvement, generalized enamel defects
66	c.[3908G>A;**4100_4101delTT]**	p.[R1303Q;F1367Cfs^*^8]	Collagen XVII is present at blister roof	Mildly affected (see Figure 1)
68	**c.[2T>A;3164delT]**	p.[?;F1055Sfs^*^11]	Not available	Severely affected (see Figure 1) and squamous cell carcinoma

*Mutations in bold have not been reported before*.

* *if only one mutation mentioned, then homozygous*.

### Novel *COL17A1* Mutations and Genotype-Phenotype Correlations

**Figure 2:**
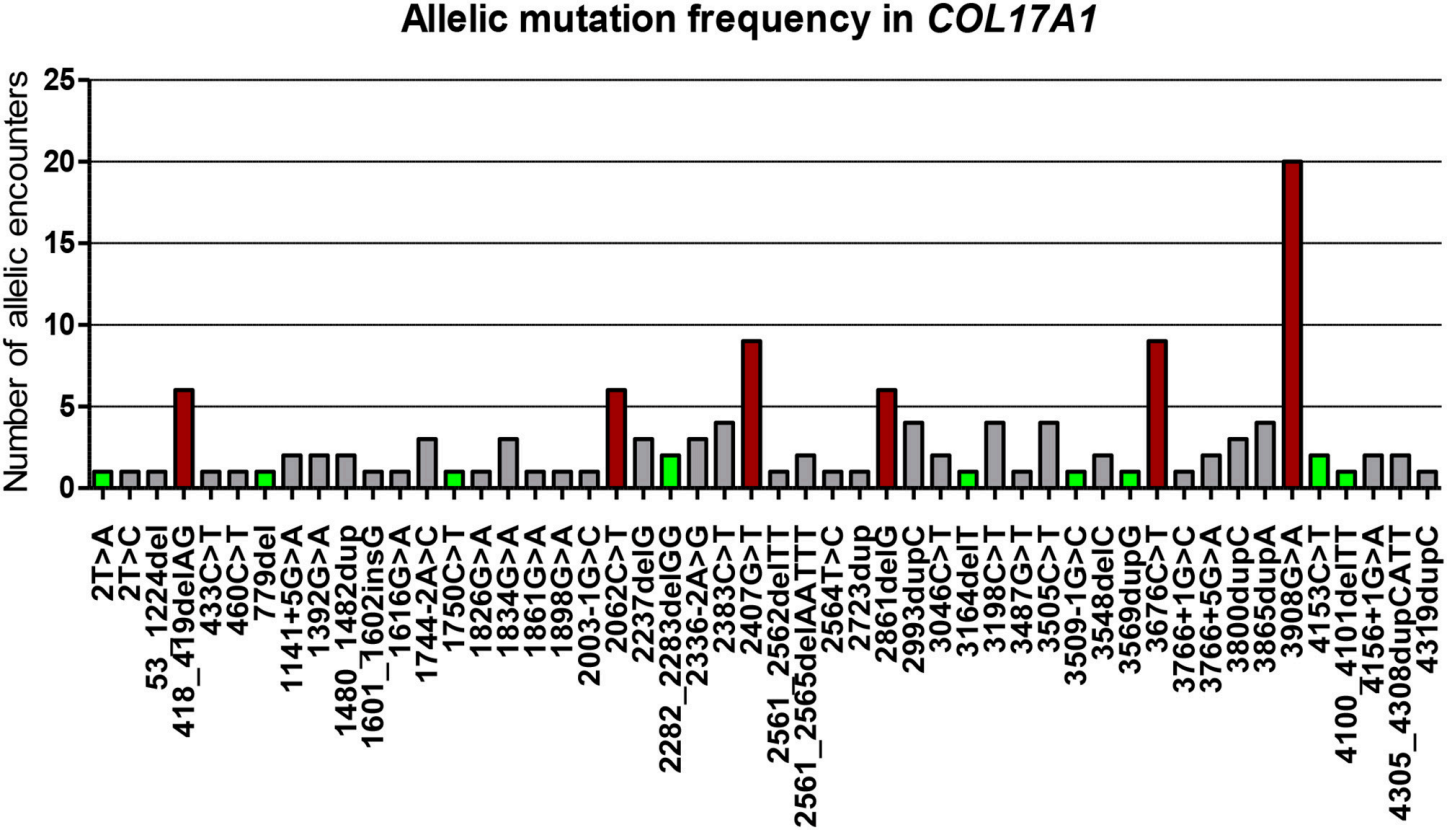
Illustration of all 50 distinct mutations (both previously published and not) and their allelic mutation frequency, included in the article. The mutations have been sorted based on their position on the *COL17A1* gene. Mutations are at cDNA levels. Novel mutations are highlighted in green squares and the most frequent are shown in dark red squares. The Y axis represents numbers of allelic encounters in our cohort.

This study contributes to the *COL17A1* mutational database with 9 mutations that, to the best of our knowledge, have not been reported before (Table 1, Figure 2). These include two nonsense mutations c.1750C > T, p.R584^*^ and c.4153C > T, p.Q1385^*^, five frameshift mutations, one mutation of the translation initiation codon, and one splice site mutation. Several of these cases demonstrated interesting genotype-phenotype correlations or clinical course of the disease and will be highlighted here.

**Figure 3:**
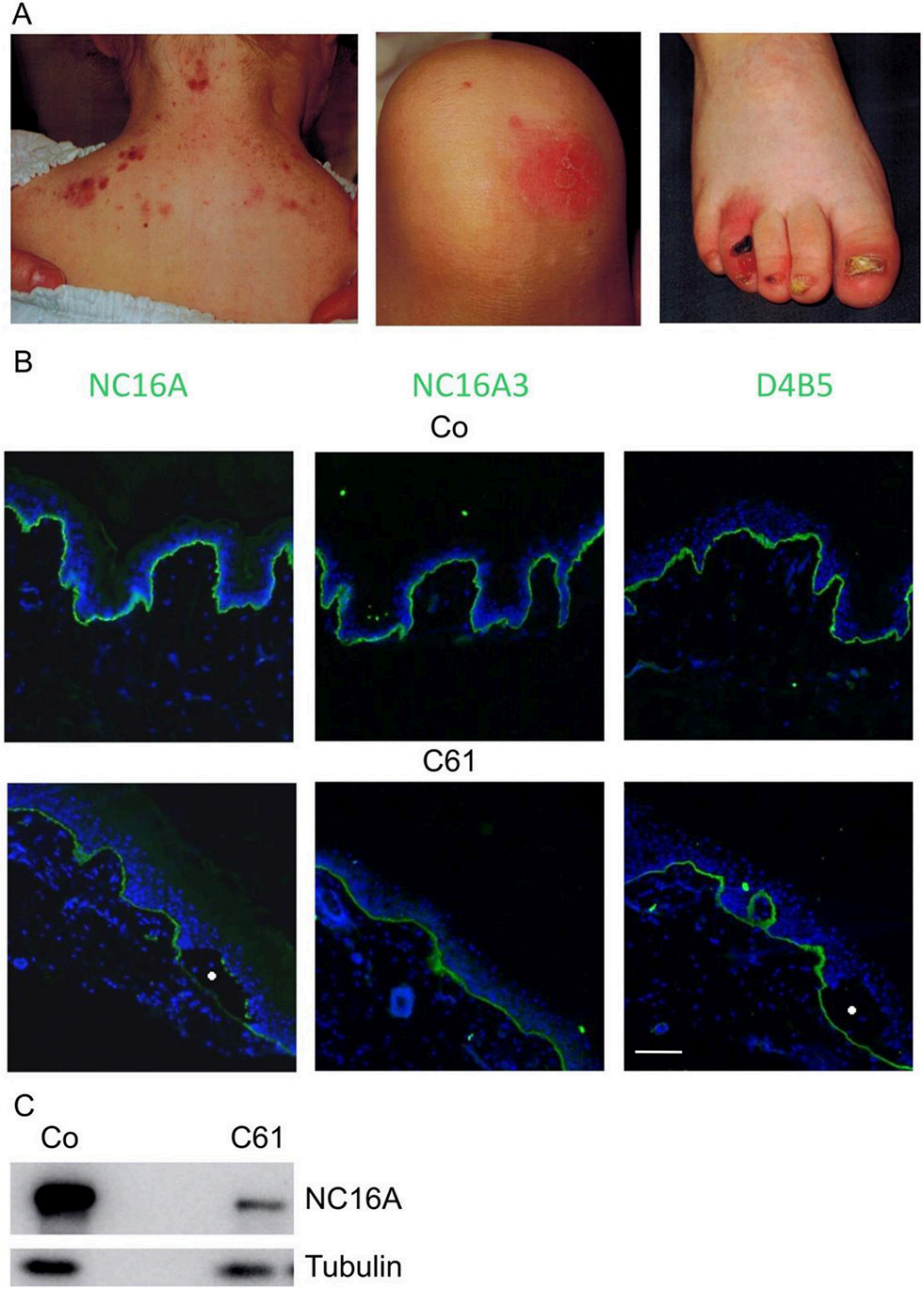
Case 61, a 2 y-old female JEB patient, mildly affected. **(A)** Clinical manifestations include skin blisters on the upper posterior thorax, erosions on the lower limb and nail dystrophy on lower limbs. **(B)** Immunofluorescence antigen mapping performed with domain-specific antibodies against collagen XVII (NC16A, NC16A3) and collagen IV (D4B5) on normal control human skin (Co, upper pictures) and case 61 (C61, lower pictures) with JEB. The antibodies used were NC16A rabbit polyclonal (Abcam), NC16A3 mouse monoclonal (Medimabs), and D4B5 mouse monoclonal for collagen IV (Abcam). Collagen XVII and collagen IV appear in green and nuclei appear in blue. Blisters are marked with circles. **(C)** Immunoblot analysis with lysates from control normal keratinocytes and from Case 61 with NC16A and antibodies to tubulin, as a loading control. Scale bar for all panels 100 μm.

Case 61 was a female patient suffering from mild skin blistering since birth. Epidermolysis bullosa simplex was suspected and several candidate genes analyzed, but no mutations were found. Clinically, skin blisters and erosions were present on the face, upper posterior thorax, and lower limbs (Figure 3). As the girl grew older, nails became dystrophic and were progressively lost, suggesting the diagnosis of JEB. She was found to be compound heterozygous for the *COL17A1* mutations c.1750C > T, p.R584^*^ and c.3509-1G > C. To the best of our knowledge both mutations were previously unreported. The mutation c.3509-1G > C is located in intron 49 of *COL17A1* and alters the conserved acceptor splice site. RT-PCR and sequencing of the RNA extracted from keratinocytes of the patient revealed that an alternative splice site was used, 33 nucleotides downstream in the middle of exon 50, thus restoring the reading frame and allowing the synthesis of an eleven amino acids shorter truncated polypeptide. Intriguingly, in immunofluorescence mapping, the immunoreactivity for collagen XVII with the commercial monoclonal antibody NC16A3 (Abcam) was comparable to the control skin (Figure 3B). The polyclonal sera NC16A (17) demonstrated reduced immunoreactivity in the skin of the patient and a decreased amount of collagen XVII in lysates from patient's keratinocytes as compared to the controls (Figure 3B), explaining the mild phenotype.

Case 66 was a 22-year-old male who presented with acral blistering that had started at the age of 16 and was induced by sport activities. Additionally, he displayed nail dystrophy (Figures 1A–C). Immunofluorescence microscopy demonstrated a broadened staining pattern of type XVII collagen and no skin split. Clinical exome sequencing demonstrated that he was compound heterozygous for the *COL17A1* mutations c.3908G>A, and the frame shift deletion c.4100_4101delTT, p.F1367Cfs^*^8, that truncates the last 130 amino acids in the C-terminus of the α1(XVII)-chain.

The severe clinical picture of generalized intermediate JEB is illustrated in case 68 from our cohort, a 45-year-old male, compound heterozygous for the previously unpublished mutation in the translation initiation codon, c.2T>A, p.?, and the also novel frame-shift deletion c.3164delT, p.F1055Sfs^*^11. Generalized blistering resulted in multiple chronic non-healing wounds at sites of permanent trauma (Figures 1D,E); on such a wound on the lower left leg, he developed a squamous cell carcinoma at the age of 44 (Figure 1F). Although initially the histological aspect was that of a verrucous carcinoma, recurrence occurred rapidly with local invasion, necessitating amputation. This is the only patient of this cohort who developed squamous cell carcinoma during the observation period. Squamous cell carcinoma has been reported in JEB patients from The Netherlands (18), but the precise risk for developing this complication in JEB remains unknown.

### Stratification of *COL17A1* Mutations

Stratified medicine, and in particular stratification of mutations, focuses on establishing a therapeutic approach tailored to the specific needs of a patient. Therefore, determining the most prevalent mutations and addressing their consequences is a practical and feasible concept, with previous work in cystic fibrosis supporting this aim (19). Currently, no international database of patients with JEB and *COL17A1* mutations is available, although many countries have established registries with clinical, molecular and follow up data for EB patients. Over 100 distinct *COL17A1* mutations have been reported in the literature (HGMD professional as of 1/15/2018) including 24 nonsense mutations, 14 missense mutations, 24 splicing mutations, 27 small deletions, 20 small insertions, one small indel, one gross deletion, and one gross insertion. Even though *COL17A1* is the only gene involved, phenotypes may differ in severity, so that mutations leading to complete loss of type XVII collagen will result in a more severe phenotype as compared to mutations that allow some degree of protein synthesis and function.

In the past 15 years we have systematically performed molecular diagnostics for EB employing immunofluorescence mapping and mutation detection (13, 15). Altogether, we identified 68 patients with JEB with *COL17A1* mutations, with a mean of about 5 new cases per year.

Fifty distinct *COL17A1* mutations were identified (Table 2). Evaluation and stratification of these mutations was performed based on the number of allelic encounters and mutation type (Figure 2). As shown in Figure 2, most mutations are unique, occurring in single cases / families and only few are recurrent. Approximately 33% of *COL17A1* mutant alleles are nonsense mutations, and another 33% are frame shift mutations leading to formation of premature termination codons (PTC). Splice site mutations account for 10% of mutant alleles.

**Table 2:** Cohort description with position of mutations on *COL17A1* gene and proposed treatment based on the type of mutation.

**COL17A1 mutations cDNA**	**Location**	**Mutation protein**	**Proposed treatment**	**Comment**
2T>A	2	?	Gene therapy	
2T>C	2	?	Gene therapy	
53_1224del	3	?	Gene therapy	
418_419delAG	8	S140^*^	Readthrough	
433C>T	8	R145^*^	Readthrough	
460C>T	8	R154^*^	Readthrough	
779del	11	P260Qfs^*^32	Gene therapy	
1141+5G>A	IVS14	Residual expression of full-length collagen XVII due to leaky splice site	Symptom reliefing therapies	Moderate disease severity
1392G>A	17	W464^*^	Readthrough	
1480_1482dup	18	K494dup	Symptom reliefing therapies	Moderate disease severity
1601_1602insG	18	S534Efs^*^10	Exon 18 in frame skipping	
1616G>A	18	G539E	Symptom reliefing therapies	Moderate disease severity
1744-2A>C	IVS20	In frame skipping of exons 21 and 22 or partial skipping of exon 21 leading to residual expression of collagen XVII	Symptom reliefing therapies	Moderate disease severity
1750C>T	21	R584^*^	Readthrough	
1826G>A	22	G609D	Symptom reliefing therapies	Moderate disease severity
1834G>A	22	G612R	Symptom reliefing therapies	Moderate disease severity
1861G>A	23	G621S	Symptom reliefing therapies	Moderate disease severity
1898G>A	23	G633D	Symptom reliefing therapies	Moderate disease severity
2003-1G>C	IVS24	?	Gene therapy	
2062C>T	26	R688^*^	Readthrough	
2237delG	30	G746Afs^*^53	Exon 30 in frame skipping	
2282_2283delGG	31	G761Dfs^*^40	Exon 31 in frameskipping	
2336-2A>G	IVS31	In frame skipping of exon 32 leading to expression of truncated protein	Symptom reliefing therapies	Moderate disease severity
2383C>T	33	R795^*^	Readthrough	
2407G>T	34	G803^*^	Readthrough	
2561_2565delAATTT	37	N854Tfs^*^109	Exon 37 in frame skipping	
2561_2562delTT	37	N854Ifs^*^110	Exon 37 in frame skipping	
2564T>G	37	L855^*^	Readthrough	
2723dup	40	G909Rfs^*^56	Exon 40 in frame skipping	
2861delG	43	G954Afs^*^112	Exon 43 in frame skipping	
2993dupC	44	G999Wfs^*^22	Exon 44 in frame skipping	
3046C>T	45	Q1016^*^	Readthrough	
3164delT	46	F1055Sfs^*^11	Exon 46 in frame skipping	
3198C>T	46	Residual expression of full-length collagen XVII due to leaky splice site	Symptom reliefing therapies	Moderate disease severity
3487G>T	49	E1163^*^	Readthrough
3505C>T	49	R1169^*^	Readthrough	
3509-1G>C	IVS49	In frame skipping of part of exon 50 leading to truncated protein expression	Symptom reliefing therapies	Moderate disease severity
3548delC	50	P1183Rfs^*^68	Exon 50 in frame skipping	
3569dupG	50	N1191Qfs^*^51	Exon 50 in frame skipping	
3676C>T	51	R1226^*^	Readthrough	Recurrent mutation
3766+1G>C	IVS51	Out of frame and absence of collagen XVII	Gene therapy	
3766+5G>A	IVS51	Absence of collagen XVII	Gene therapy	
3800dupC	52	G1268Rfs^*^25	Exon 52 in frame skipping	
3865dupA	52	S1289Kfs^*^4	Exon 52 in frame skipping	
3908G>A	52	R1303Q	Symptom reliefing therapies	Late onset and moderate disease severity
4153C>T	52	Q1385^*^	Readthrough	
4100_4101delTT	52	F1367Cfs^*^8	Exon 52 in frame skipping	
4156+1G>A	IVS52	In frame skipping of exon 52	Symptom reliefing therapies	Moderate disease severity
4305_4308dupCATT	54	Q1437Hfs^*^19	Symptom reliefing therapies	Moderate disease severity
4319dupC	54	G1441Wfs^*^14	Symptom reliefing therapies	Moderate disease severity

* *stands for a premature termination codon*.

In this cohort, the most frequent recurrent mutation was c.3908G > A, p.R1303Q detected in 16.17% of cases and 14.70% of the mutant alleles (in 9 patients in a homozygous state and in 2 patients in heterozygous manner), respectively. This missense mutation changes the amino acid arginine with glutamine and is located in the fourth non-collagenous domain (NC4) of the α1(XVII)-chain, which is part of the putative laminin-332 binding region in type XVII collagen (20). Secondary protein structure analysis and predicted changes in the structural integrity within this binding region suggested that these modifications result in abnormal laminin-332 binding (21). This mutation also appears to hamper the physiological C-terminal cleavage of type XVII collagen. Consequently, non-cleaved type XVII collagen ectodomain remnants induce the aberrant deposition of laminin-332 in the extracellular matrix (12, 22). Clinically, p.R1303Q is associated with a mild, Kindler-syndrome-like phenotype, manifesting with late onset skin blistering, progressive skin atrophy and sclerosis, loss of dermatoglyphics, scarring, and nail anomalies. Typically, type XVII collagen is deposited at the dermal-epidermal junction in a broad irregular pattern (13, 16).

Second most common mutation was c.2407G > T, representing 6.61% of the mutant alleles in 10.29% of cases (in 5 patients in a heterozygous state and in 2 homozygous patients). This mutation located in exon 34, where it converts a glycine residue to a premature termination codon (PTC), p.G803^*^,was also frequent in patients with Austrian (18) and Finish background (23). With the same allele frequency there was also c. 3676C > T, found in 11.76% of patients. It also translates into a PTC, p.R1226^*^. This recurrent mutation has also been previously reported in Dutch population (24) and leads to nonsense mediated mRNA decay, demonstrated by absence of full length collagen XVII (12, 25). Next, we found c.2062C > T, p.R688^*^, c.2861delG, p.G954Afs^*^112 and 418_419delAG, p.S140^*^ to represent 4.41% of the mutant alleles in 4.31% of our patients. All these null-mutations lead to absence of type XVII collagen and generalized intermediate JEB (previously known as generalized atrophic benign epidermolysis bullosa (GABEB) and later as non-Herlitz JEB). This most severe *COL17A1*-associated phenotype was the first to be recognized (12, 17, 22). The main clinical manifestation is represented by mechanically induced skin blistering that starts at birth, is generalized and persists lifelong, without spontaneous improvement. Repeated wound and healing episodes result in skin scarring and atrophy. Nails become dystrophic and are progressively lost, as are scalp and body hairs. Teeth exhibit amelogenesis imperfecta which leads to excessive and premature caries and loss of dentition.

Such stratification of mutations endorses the high frequency of nonsense mutations or frame-shift leading to premature termination codons and the need for therapy of JEB patients. Moreover, the recurrence rates gives perspective to the possible similarities between different cultural backgrounds or to the extent in which external factors may impact this disease.

## Discussions

### Consequences of *COL17A1* Mutations and Potential Therapeutic Strategies

Potential therapeutic strategies emerge from analysis of the mutations, their consequences and from the resulting phenotypes (Table 2). Patients with acral or late onset JEB, like our cases 61 and 66, have limited disease burden and can be treated symptomatically. In contrast, severely affected patients, like our case 68 require effective therapeutic strategies for their chronic wounds. There is also no existing treatment for other disease manifestations, such as: (i) hair loss, which is definitive; (ii) nail dystrophy or loss, which are regarded as cosmetic problems; (iii) amelogenesis imperfecta leading to increased caries and premature destruction of teeth, which require careful and sustained dental care and treatment. The analysis of genotype-phenotype correlations in patients the JEB suggests that 12–25% of the normal type XVII collagen levels are sufficient to provide a certain degree of skin stability and consequently ameliorate the phenotype (3).

Taking into account the large number of *COL17A1* PTC-mutations, therapies to restore the mutant gene seem to be appropriate in this EB subtype. In our study 33% of mutations are nonsense mutations. These relatively high figures render the thoroughfare for an efficient therapeutic approach directed against these particular types of mutations. *Ex-vivo* gene therapy, where the defective gene is corrected, transduced in cells and later grafted to the patient is highly complex and expensive. As an alternative approach, RNA trans-splicing was developed and showed expression of the replaced exon 52 in up to 61% assayed cells *in vitro* (26).

Revertant mosaicism is frequent in patients with JEB-*COL17A1* (18), but GMP-cultivation and expansion of revertant epidermal stem cells is not yet broadly available. Because type XVII is solely synthetized by keratinocytes and must be correctly assembled as a transmembrane protein in a supramolecular complex, fibroblast / mesenchymal stem cell therapies and protein therapy are not feasible.

Type XVII collagen has a modular collagenous structure, and *COL17A1* has 54 out of 56 exons in-frame, except of the first and last. In-frame truncations seem to be tolerated, and account for the presence of molecules which are stable and partially functional, such in our case 61 and in other cases reported in the literature (27, 28). Thus, antisense-mediated skipping of exons with PTCs might also be an alternative strategy for *COL17A1* mutations (29).

Nevertheless, drug-based therapeutic strategies that aim to modify either the synthesis of protein by inducing readthrough or the mechanisms that lay the ground for RNA translation might be easier available. Suppression of stop codons and enabling readthrough in translation can be effective for several genetic disorders, including EB (24, 27, 30, 31).

Readthrough therapy modifies the response of the translational ribosome to nonsense mutations, by allowing a near-cognate aminoacyl tRNA to step into the spot on the stop codon and thus proceeding toward protein synthesis and allowing a functional protein to be produced. In order to restore the function of nonsense mutations carrying genes several compounds have been identified in the last decades. The PTCs are normally recognized by a mechanism called nonsense-mediated mRNA decay (NMD), which tries to eliminate them in order to reduce errors in the expression of a particular gene. NMD efficiency can influence the response of readthrough by several factors (32). Particularly, when some key factors in NMD pathway are inhibited, such as ATP dependent RNA helicase upframeshift 1 (UPF1) and upframeshift 2 (UPF2), a better function of the gene implicated in the pathology of cystic fibrosis was noticed (32). On the contrary, in recessive dystrophic epidermolysis bullosa, it has been shown that a better response to readthrough was associated with an increase in the factor UPF1 in all cells treated, results which support that the more stable this UPF1 factor is, the more inhibited NMD pathway is (33). Another factor that can be limiting in the response of readthrough could be the PTC bearing transcripts of patients. These may vary among individuals and could be beneficial to assess before treatment (32).

## Conclusions

We report here nine previously unpublished mutations, which will enhance the already established database with *COL17A1* mutations. The additionally reported genotype-phenotype correlations will help in diagnosis and genetic counseling, thus providing advantage in the clinical setting. The stratification of the mutations and their consequences should also provide insights for mutation analysis strategies and potential therapies.

As far as therapeutic strategies are regarded for *COL17A1* PTC-mutations, readthrough compounds and exon skipping-approaches seem to be reliable and aim at increasing stability of the skin, wound healing and improving the quality of life. Progress still needs to be made for the other cutting-edge therapeutic possibilities.

## Author Contributions

CH has molecularly characterized the cohort of patients and has written the final version of the manuscript. IC has analyzed the data, performed the molecular analyses in patient 61, the characterization of the cell lines and has drafted the manuscript and figures. YH has analyzed data. RC has contributed with the phenotypes analysis. All authors have read and corrected the manuscript.

### Conflict of Interest Statement

The authors declare that the research was conducted in the absence of any commercial or financial relationships that could be construed as a potential conflict of interest.

## Abbreviations

EB: epidermolysis bullosa
JEB: junctional epidermolysis bullosa
PTC: premature termination codon
NMD: nonsense mediated decay.

## References

[1] Fine J-D Bruckner-Tuderman L Eady RAJ Bauer EA Bauer JW Has C. Inherited epidermolysis bullosa: updated recommendations on diagnosis and classification. J Am Acad Dermatol. (2014) 70:1103–26. 10.1016/j.jaad.2014.01.903 24690439

[2] Walko G Castañón MJ Wiche G Molecular architecture and function of the hemidesmosome. Cell Tissue Res. (2015) 360:529–44. 10.1007/s00441-015-2216-6 26017636 PMC4452579

[3] Has C Nyström A Saeidian AH Bruckner-Tuderman L Uitto J Epidermolysis bullosa: Molecular pathology of connective tissue components in the cutaneous basement membrane zone. Matrix Biol. (2018) 71–72:313–29. 10.1016/j.matbio.2018.04.001 29627521

[4] Natsuga K Watanabe M Nishie W Shimizu H. Life before and beyond blistering: the role of collagen XVII in epidermal physiology. Exp Dermatol. (2018). [Epub ahead of print]. 10.1111/exd.13550 29604146

[5] Watanabe M Natsuga K Nishie W Kobayashi Y Donati G Suzuki S. Type XVII collagen coordinates proliferation in the interfollicular epidermis. eLife (2017) 6:e26635. 10.7554/eLife.26635 28693719 PMC5505703

[6] Hiroyasu S Colburn ZT Jones JCR. A hemidesmosomal protein regulates actin dynamics and traction forces in motile keratinocytes. FASEB J. (2016) 30:2298–310. 10.1096/fj.201500160R 26936359 PMC4871795

[7] Matsumura H Mohri Y Binh NT Morinaga H Fukuda M Ito M. Hair follicle aging is driven by transepidermal elimination of stem cells via COL17A1 proteolysis. Science (2016) 351:aad4395. 10.1126/science.aad4395 26912707

[8] Tanimura S Tadokoro Y Inomata K Binh NT Nishie W Yamazaki S. Hair follicle stem cells provide a functional niche for melanocyte stem cells. Cell Stem Cell (2011) 8:177–87. 10.1016/j.stem.2010.11.029 21295274

[9] Uitto J McGrath JA Rodeck U Bruckner-Tuderman L Robinson EC. Progress in epidermolysis bullosa research: toward treatment and cure. J Invest Dermatol. (2010) 130:1778–84. 10.1038/jid.2010.90 20393479

[10] Hirsch T Rothoeft T Teig N Bauer JW Pellegrini G De Rosa L. Regeneration of the entire human epidermis using transgenic stem cells. Nature (2017) 551:327–32. 10.1038/nature24487 29144448 PMC6283270

[11] Siprashvili Z Nguyen NT Gorell ES Loutit K Khuu P Furukawa LK. Safety and wound outcomes following genetically corrected autologous epidermal grafts in patients with recessive dystrophic epidermolysis bullosa. JAMA (2016) 316:1808–17. 10.1001/jama.2016.15588 27802546

[12] Kroeger J Hoppe E Galiger C Has C Franzke C-W. Amino acid substitution in the C-terminal domain of collagen XVII reduces laminin-332 interaction causing mild skin fragility with atrophic scarring. Matrix Biol J Int Soc Matrix Biol. (2018). 10.1016/j.matbio.2018.10.003 30316981

[13] Has C Küsel J Reimer A Hoffmann J Schauer F Zimmer A. The position of targeted next-generation sequencing in epidermolysis bullosa diagnosis. Acta Derm Venereol. (2018) 98:437–40. 10.2340/00015555-2863 29242947

[14] Maier K He Y Esser PR Thriene K Sarca D Kohlhase J. Single amino acid deletion in kindlin-1 results in partial protein degradation which can be rescued by chaperone treatment. J Invest Dermatol. (2016) 136:920–9. 10.1016/j.jid.2015.12.039 26827766

[15] Has C He Y. Research techniques made simple: immunofluorescence antigen mapping in epidermolysis bullosa. J Invest Dermatol. (2016) 136:e65–71. 10.1016/j.jid.2016.05.093 27342035

[16] Schäcke H Schumann H Hammami-Hauasli N Raghunath M Bruckner-Tuderman L. Two forms of collagen XVII in keratinocytes. A full-length transmembrane protein and a soluble ectodomain. J Biol Chem. (1998) 273:25937–43. 10.1074/jbc.273.40.25937 9748270

[17] Yuen WY Pas HH Sinke RJ Jonkman MF. Junctional epidermolysis bullosa of late onset explained by mutations in COL17A1. Br J Dermatol. (2011) 164:1280–4. 10.1111/j.1365-2133.2011.10359.x 21466533

[18] Darling TN McGrath JA Yee C Gatalica B Hametner R Bauer JW. Premature termination codons are present on both alleles of the bullous pemphigoid antigen 2/type XVII collagen gene in five Austrian families with generalized atrophic benign epidermolysis bullosa. J Invest Dermatol. (1997) 108:463–8. 10.1111/1523-1747.ep12289718 9077475

[19] Bobadilla JL Macek M Fine JP Farrell PM. Cystic fibrosis: a worldwide analysis of CFTR mutations–correlation with incidence data and application to screening. Hum Mutat. (2002) 19:575–606. 10.1002/humu.10041 12007216

[20] Tasanen K Tunggal L Chometon G Bruckner-Tuderman L Aumailley M. Keratinocytes from patients lacking collagen XVII display a migratory phenotype. Am J Pathol. (2004) 164:2027–38. 10.1016/S0002-9440(10)63762-5 15161638 PMC1615787

[21] Has C Kiritsi D Mellerio JE Franzke C-W Wedgeworth E Tantcheva-Poor I. The missense mutation p.R1303Q in type XVII collagen underlies junctional epidermolysis bullosa resembling Kindler syndrome. J Invest Dermatol. (2014) 134:845–9. 10.1038/jid.2013.367 24005051

[22] Nishimura M Nishie W Shirafuji Y Shinkuma S Natsuga K Nakamura H. Extracellular cleavage of collagen XVII is essential for correct cutaneous basement membrane formation. Hum Mol Genet. (2016) 25:328–39. 10.1093/hmg/ddv478 26604146

[23] Gatalica B Pulkkinen L Li K Kuokkanen K Ryynänen M McGrath JA. Cloning of the human type XVII collagen gene (COL17A1), and detection of novel mutations in generalized atrophic benign epidermolysis bullosa. Am J Hum Genet. (1997) 60:352–65. 9012408 PMC1712405

[24] Pasmooij AMG Pas HH Jansen GHL Lemmink HH Jonkman MF. Localized and generalized forms of blistering in junctional epidermolysis bullosa due to COL17A1 mutations in the Netherlanhds. Br J Dermatol. (2007) 156:861–70. 10.1111/j.1365-2133.2006.07730.x 17263807

[25] McGrath JA Gatalica B Christiano AM Li K Owaribe K McMillan JR. Mutations in the 180-kD bullous pemphigoid antigen (BPAG2), a hemidesmosomal transmembrane collagen (COL17A1), in generalized atrophic benign epidermolysis bullosa. Nat Genet. (1995) 11:83–6. 10.1038/ng0995-83 7550320

[26] Koller U Wally V Mitchell LG Klausegger A Murauer EM Mayr E. A novel screening system improves genetic correction by internal exon replacement. Nucleic Acids Res. (2011) 39:e108. 10.1093/nar/gkr465 21685452 PMC3167625

[27] Yancey KB Hintner H. Non-herlitz junctional epidermolysis bullosa. Dermatol Clin. (2010) 28:67–77. 10.1016/j.det.2009.10.008 19945618

[28] Hintner H Wolff K. Generalized atrophic benign epidermolysis bullosa. Arch Dermatol. (1982) 118:375–84. 10.1001/archderm.1982.01650180009008 7092249

[29] Lincoln V Cogan J Hou Y Hirsch M Hao M Alexeev V. Gentamicin induces LAMB3 nonsense mutation readthrough and restores functional laminin 332 in junctional epidermolysis bullosa. Proc Natl Acad Sci USA. (2018) 115:E6536–45. 10.1073/pnas.1803154115 29946029 PMC6048497

[30] Huber M Floeth M Borradori L Schäcke H Rugg EL Lane EB. Deletion of the cytoplasmatic domain of BP180/collagen XVII causes a phenotype with predominant features of epidermolysis bullosa simplex. J Invest Dermatol. (2002) 118:185–92. 10.1046/j.0022-202x.2001.01617.x 11851893

[31] Bruckner-Tuderman L Has C. Disorders of the cutaneous basement membrane zone–the paradigm of epidermolysis bullosa. Matrix Biol J Int Soc Matrix Biol. (2014) 33:29–34. 10.1016/j.matbio.2013.07.007 23917088

[32] Linde L Kerem B. Introducing sense into nonsense in treatments of human genetic diseases. Trends Genet TIG (2008) 24:552–63. 10.1016/j.tig.2008.08.010 18937996

[33] Kurosaki T Li W Hoque M Popp MW-L Ermolenko DN Tian B. A post-translational regulatory switch on UPF1 controls targeted mRNA degradation. Genes Dev. (2014) 28:1900–16. 10.1101/gad.245506.114 25184677 PMC4197951

